# Attitudes towards COVID-19 vaccination and intention to get vaccinated in Western Balkans: cross-sectional survey

**DOI:** 10.1093/eurpub/ckad066

**Published:** 2023-04-29

**Authors:** Vida Jeremic Stojkovic, Smiljana Cvjetkovic, Janko Jankovic, Stefan Mandic-Rajcevic, Sanja Matovic Miljanovic, Aleksandar Stevanovic, Aleksandra Jovic Vranes, Zeljka Stamenkovic

**Affiliations:** Faculty of Medicine, University of Belgrade, Department of Humanities, Belgrade, Serbia; Faculty of Medicine, University of Belgrade, Department of Humanities, Belgrade, Serbia; Faculty of Medicine, University of Belgrade, Institute of Social Medicine, Belgrade, Serbia; Faculty of Medicine, University of Belgrade, Institute of Social Medicine, Belgrade, Serbia; Euro Health Group A/S, Denmark and Regional office Serbia, Belgrade, Serbia; Faculty of Medicine, University of Belgrade, Institute of Social Medicine, Belgrade, Serbia; Faculty of Medicine, University of Belgrade, Institute of Social Medicine, Belgrade, Serbia; Faculty of Medicine, University of Belgrade, Institute of Social Medicine, Belgrade, Serbia

## Abstract

**Background:**

Introduction of vaccines against COVID-19 has not encountered expected acceptance. The uptake of COVID-19 vaccines in Western Balkans countries is lagging behind the European Union average. The aim of our study was to assess the intention to get vaccinated against COVID-19 in the population of unvaccinated adult citizens of five Western Balkans countries, and to explore factors that influence the vaccination intention.

**Methods:**

Cross-sectional study was conducted in the period from July to October 2021. The questionnaire was shared through online social media. Intention to get vaccinated against COVID-19 was measured by a single item assessing the likelihood of getting vaccinated on a 5-points Likert scale. Linear regressions were conducted with socio-demographic characteristics, presence of chronic diseases and attitudes towards COVID-19 vaccination as independent factors.

**Results:**

The largest proportion of unvaccinated respondents willing to get vaccinated in the future was observed in Montenegro and Albania (40.4% in each country), while in the Serbian sample, the willingness to get vaccinated was the lowest (22.6%). Socio-demographic characteristics were not significantly associated with the intention to get vaccinated against COVID-19 in most of the countries. In Albania, Bosnia and Herzegovina, North Macedonia and Serbia the strongest determinant of COVID-19 vaccination intention was the higher sense of social responsibility.

**Conclusions:**

Vaccination interventions and campaigns aiming to improve the COVID-19 vaccine uptake should be focussed on specific set of factors in each country, appealing to social responsibility as most prevalent determinant of vaccination intention in Western Balkans.

## Introduction

Although vaccination is the most effective preventive measure against diseases and cost-effective public health intervention, vaccine uptake is suboptimal in many countries worldwide. Numerous studies have explored factors that influence people’s decision to get vaccinated, using different methodologies and presenting different strength of evidence.^1^^,^^2^ In developed countries, issues of access and vaccine availability are of less concern, while psychological, social and contextual factors are defined as main drivers of vaccine uptake.^1^ More recent global survey conducted in 23 countries shows that, across all countries, vaccine hesitancy is associated with a lack of trust in COVID-19 vaccine safety and science, and scepticism about its efficacy.^3^ In addition, vaccine acceptance or refusal is context-dependent, so the influence of cultural, political, historical and economic factors should be taken into account.^4^ Introduction of vaccines against COVID-19, although eagerly awaited by many countries, has not encountered expected acceptance. It could be assumed that factors influencing individuals’ choice on whether to get vaccinated against COVID-19 are similar to factors of routine vaccination behaviour, yet the circumstances are different. Political factors, erosion of societal trust, greater uncertainty and overabundance of information complicate people’s comprehension and response to the policy measures to the pandemic, resulting in lower vaccine acceptance rates.^4^ Research conducted so far suggests that acceptance of a COVID-19 vaccine is far from universal.^5–9^ Several systematic reviews were conducted^10–12^ aiming to summarize factors correlated with COVID-19 vaccination acceptance. According to them, some of the most pronounced factors of vaccination intention are vaccine-related perceptions (of vaccine efficacy and vaccine safety), perceived risk of COVID-19, institutional trust, sense of collective responsibility, information environment and socio-demographic factors.

The uptake of COVID-19 vaccines in Western Balkans countries is lagging behind the European Union (EU) average. According to the last available official data the proportion of population fully vaccinated (with two doses) against COVID-19 is 40% in North Macedonian, 44% in Albania, 48% in Serbia, 45% in Montenegro and 26% in Bosnia and Herzegovina, which is far below the proportion of 73% in the EU.^13^ Higher uptake of COVID-19 vaccines in some EU countries could be partially explained by introduction of mandatory vaccination for some categories of citizens. However, a limited number of studies explored reasons for lower COVID-19 vaccination acceptance in Eastern European and Balkan countries.^14^ Understanding factors that influence vaccine uptake in those countries is critical for development of effective vaccination promotion strategies for Western Balkans. Accordingly, the aim of our study was to assess the intention to get vaccinated against COVID-19 in the population of unvaccinated adult citizens of five Western Balkans countries (Albania, Bosnia and Herzegovina, Montenegro, North Macedonia and Serbia), and to explore socio-demographic and attitudinal factors that influence the vaccination intention.

## Methods

### Sampling and procedure

As part of a larger project investigating COVID-19 related vaccine hesitancy in Western Balkans, this cross-sectional study was conducted in the period from July to October 2021. Adult citizens over the age of 18 from the five Western Balkans countries (Albania, Bosnia and Herzegovina, North Macedonia, Montenegro and Serbia) were included. Convenience, non-probability sampling was applied. Data were collected by means of the online questionnaire, using the SurveyMonkey platform, which automatically saves digital responses to a database. The questionnaire was shared through widely used online social media (Facebook and Instagram), using targeted posting method. Individuals could access the questionnaire link by clicking on the post. It took ∼15–20 min to complete the questionnaire. Intention to get vaccinated was analyzed in the sub-sample of unvaccinated respondents, since vaccinated already manifested their intention.

### Study instrument and measures

The questionnaire was developed for the purpose of this study based on the literature.^15–17^ It was originally developed in English, and country experts were engaged to translate and adapt it to the language of each country participating in the project. The questionnaire consisted of four parts:

1. The primary outcome—‘intention to get vaccinated against COVID-19’ was measured by a single item assessing the likelihood of getting vaccinated on a 5-points Likert scale (ranging from 1—extremely unlikely to 5—extremely likely);
2. Socio-demographic characteristics included six items: gender, age, education level, employment status (employed/self-employed/unemployed), financial status (very good/good/average/bad/very bad) and religiosity (yes/no);
3. Existence of the chronic health conditions was assessed by the single question ‘Do you suffer from any chronic disease?’ with the binary (yes/no) response;
4. Seven short scales measuring different attitudes related to COVID-19 vaccination, on a 5-point agreement Likert scales (ranging from 1 ‘strongly disagree’ to 5 ‘strongly agree’). The total score for each scale was calculated by summing the responses to all items, and dividing that sum with the number of items. Items with negative connotation were reversely coded when calculating the total scores. The total score range for each scale was divided in four quartiles: 1–1.99 (highly negative), 2–2.99 (moderately negative), 3–3.99 (moderately positive) and 4–5 (highly positive). Items of each attitude scale are presented in the Supplementary table S1.
  – Attitudes towards vaccine efficacy (one-factor scale, five items, *α* ranging from 0.52 to 0.93 across country samples).
  – Attitudes towards vaccine safety (one-factor scale, six items, *α* ranging from 0.53 to 0.91 across country samples).
  – Attitudes towards compulsory vaccination (one-factor scale, two items, *α* ranging from 0.70 to 0.91 across country samples).
  – Attitudes towards danger of COVID-19 disease (one-factor scale, five items, *α* ranging from 0.85 to 0.89 across country samples).
  – Attitudes towards COVID-19 susceptibility (one-factor scale, three items, *α* ranging from 0.70 to 0.75 across country samples).
  – Trust in societal factors (one-factor scale, six items, *α* ranging from 0.87 to 0.91 across country samples). Social responsibility (one-factor scale, three items, *α* ranging from 0.75 to 0.83 across country samples).

### Ethical considerations

The work described has been carried out in accordance with the Declaration of Helsinki, which outline the ethical principles for medical research involving human subjects. The informed consent was obtained from each participant: the introductory part of the questionnaire contained information about the purpose of the study, and required from participants to check the specified box if they agreed to participate in the study. The questionnaire was anonymous, no personally identifying data were collected. Participation in the study was voluntary, no incentives were provided to respondents.

### Statistical analysis

Descriptive statistics were used to characterize the sample and study variables. To identify determinants of intention to get vaccinated in the sub-sample of unvaccinated respondents in each country, linear regressions were conducted with socio-demographic characteristics, presence of chronic diseases and attitudes towards COVID-19 vaccination as independent factors. Variables found to be significant in univariate analysis were entered in multiple regression analysis for each country.

All analyses were performed in Statistical Package for Social Sciences for Windows, version 25 (IBM Corp., Armonk, NY) and *P *<* *0.05 was considered statistically significant.

## Results

### Socio-demographic characteristics of respondents

Overall, 1605 of respondents fully completed the questionnaire. Socio-demographic and health-related characteristics of respondents per country are presented in table 1. The average age in the whole sample was 37.52 ± 14.26, ranging from 18 to 79. Women accounted for 68.1% of respondents, while the majority had a bachelor degree (42.1%), were employed (57.1%), had an average financial situation (46.4%), declared as religious (64.4%), and did not report having any chronic disease (78.3%).

**Table 1:** Socio-demographic and health-related characteristics of respondents in five Western Balkans countries: Albania, Bosnia and Herzegovina, Montenegro, North Macedonia and Serbia

Variables	Serbia	Albania	Bosnia and Herzegovina	North Macedonia	Montenegro
Age [*M* (range)]	43.10 (19–79)	36.32 (19–70)	32.13 (18–68)	31.47 (18–71)	37.50 (18–75)
	*N* (%)	*N* (%)	*N* (%)	*N* (%)	*N* (%)
Gender					
Male	209 (35.8)	115 (37.8)	74 (33.0)	69 (25.0)	45 (20.6)
Female	374 (64.2)	189 (62.2)	150 (67.0)	207 (75.0)	173 (79.4)
Education					
High school	146 (25.0)	30 (9.9)	135 (60.3)	109 (39.9)	84 (38.9)
Bachelor degree	241 (41.3)	129 (42.4)	75 (33.5)	129 (47.3)	100 (46.3)
Master degree	121 (20.8)	112 (36.8)	13 (5.8)	28 (10.3)	22 (10.2)
Ph.D.	75 (12.9)	33 (10.9)	1 (0.4)	7 (2.6)	10 (4.6)
Employment					
Employed	373 (64.0)	193 (63.5)	95 (42.4)	126 (45.7)	129 (59.2)
Self-employed	91 (15.6)	32 (10.5)	17 (7.6)	19 (6.9)	27 (12.4)
Unemployed	119 (20.4)	79 (26.0)	112 (50.0)	131 (47.5)	62 (28.4)
Financial situation					
Very good	69 (11.8)	28 (9.2)	33 (14.7)	24 (8.7)	15 (6.9)
Good	214 (36.7)	95 (31.3)	91 (40.6)	88 (31.9)	76 (34.9)
Average	249 (42.7)	157 (51.6)	92 (41.1)	132 (47.8)	114 (52.3)
Bad	45 (7.7)	23 (7.6)	6 (2.7)	27 (9.8)	11 (5.0)
Very bad	6 (1.0)	1 (.3)	2 (0.9)	5 (1.8)	2 (.9)
Religiousness					
Yes	351 (60.2)	179 (58.9)	172 (76.8)	182 (65.9)	150 (68.8)
No	232 (39.8)	125 (41.1)	52 (23.2)	94 (34.1)	68 (31.2)
Chronic disease					
Yes	162 (27.8)	54 (17.8)	45 (21.1)	41 (14.9)	46 (21.1)
No	421 (72.2)	250 (82.2)	179 (79.9)	235 (85.1)	172 (78.9)

### Attitudes towards COVID-19 vaccination

Respondents from five surveyed countries differed in their attitudes towards COVID-19 vaccination (table 2). In all countries participants manifested moderately positive attitudes towards vaccine efficacy and safety, and expressed moderately high level of feeling susceptible to COVID-19, as well as moderately high level of societal trust. Also, in all studied countries respondents demonstrated perception of a high danger of the COVID-19 disease and a high level of social responsibility. The most positive attitude towards vaccine efficacy, vaccine safety and compulsory vaccination were found in North Macedonia. The most negative attitude towards vaccine safety and vaccine efficacy was revealed in Bosnia and Herzegovina, while the most negative attitude towards compulsory vaccination has been found in Albania. The COVID-19 disease was perceived as the most dangerous in North Macedonia, while in Bosnia and Herzegovina it was evaluated as the least hazardous. The highest degree of self-assessed susceptibility to the COVID-19 was found in Montenegro, while the lowest rate has been revealed in Albania. The highest degrees of societal trust and social responsibility were revealed in North Macedonia, while societal trust was the least pronounced in Bosnia and Herzegovina. Respondents in Albania expressed the lowest level of social responsibility.

**Table 2:** Attitudes towards COVID-19 vaccination scales’ total scores per country

Attitude scale	Albania	Bosnia and Herzegovina	Montenegro	North Macedonia	Serbia
	Mean (SD)	Mean (SD)	Mean (SD)	Mean (SD)	Mean (SD)
Attitude towards vaccine efficacy	3.30 (0.97)	3.14 (1.13)	3.39 (1.28)	3.67 (1.15)	3.34 (1.36)
Attitude towards vaccine safety	3.18 (1.06)	3.18 (1.11)	3.44 (1.22)	3.61 (1.17)	3.50 (1.26)
Attitude towards compulsory vaccination	2.04 (1.18)	2.40 (1.44)	2.77 (1.57)	3,27 (1.40)	2.97 (1.62)
Attitude towards the danger of the COVID-19 disease	3.76 (0.98)	3.54 (1.16)	3.88 (1.15)	4.25 (0.96)	3.72 (1.27)
Attitude towards COVID-19 susceptibility	3.21 (1.12)	3.35 (1.15)	3.53 (1.18)	3.44 (1.10)	3.43 (1.13)
Trust in societal factors	3.35 (1.05)	3.24 (1.10)	3.50 (1.19)	3.65 (1.06)	3.24 (1.26)
Social responsibility	3.68 (1.11)	3.69 (1.11)	3.95 (1.24)	4.12 (1.09)	3.78 (1.31)

### Intention to get vaccinated against COVID-19

Intention to get vaccinated was explored in the sub-sample of 700 respondents from the five studied countries who declared to be unvaccinated, since vaccinated respondents already manifested their positive intention. At the time of conducting the study third and consecutive doses of the vaccine were not an option.

In the total sample of unvaccinated respondents, 13.9% will certainly and 18.5% will probably get vaccinated in the future, while 20.8% were unsure. Almost half of the respondents (46.8%) probably or certainly did not intend to get vaccinated. The largest proportion of unvaccinated respondents willing to get vaccinated in the future was observed in Montenegro and Albania (40.4% in each country), while in the Serbian sample the willingness to get vaccinated was the lowest (only 22.6% reported they would probably or certainly get the vaccine in the future) (table 3).The proportion of respondents willing to get vaccinated was similar in North Macedonia (36.9%) and Bosnia and Herzegovina (39.5%).

**Table 3:** Intention to get vaccinated against COVID-19 per country

Country	Likelihood of getting vaccinated in the future					
	Certainly not	Probably not	Not sure	Probably yes	Certainly yes	Total
	*N* (%)	*N* (%)	*N* (%)	*N* (%)	*N* (%)	*N* (%)
Albania	24 (14.5)	32 (19.3)	43 (25.9)	32 (19.3)	35 (21.1)	166 (100)
Bosnia and Herzegovina	15 (10.6)	31 (22.5)	39 (27.5)	39 (27.5)	17 (12.0)	142 (100)
Montenegro	25 (25.3)	18 (18.2)	16 (16.2)	22 (22.2)	18 (18.2)	99 (100)
North Macedonia	24 (23.3)	18 (17.5)	23 (22.3)	22 (21.4)	16 (15.5)	103 (100)
Serbia	85 (44.7)	38 (20.0)	24 (12.6)	15 (7.9)	28 (14.7)	190 (100)

The results of multivariate regression analyses are presented in table 4. Socio-demographic characteristic were not significantly associated with the intention to get vaccinated against COVID-19, with the exception of Montenegro where the age of respondents was the single predictor, with younger respondents being more likely to get vaccinated. In all other studied countries (Albania, Bosnia and Herzegovina, North Macedonia and Serbia) the strongest determinant of COVID-19 vaccination intention was the higher sense of social responsibility. Positive attitude towards vaccine efficacy was significant determinant in Serbia, Bosnia and Herzegovina and Albania, while positive attitude towards vaccine safety and higher appreciation of COVID-19 as a dangerous disease significantly predicted intention in Bosnia and Herzegovina and Albania. Respondents in Serbia who felt more susceptible to COVID-10 were more willing to get vaccinated.

**Table 4:** Predictors of intention to get vaccinated against COVID-19 in the future

Predictors	Standardized coefficients beta				
	Serbia	North Macedonia	Montenegro	Bosnia and Herzegovina	Albania
Gender	−0.07	0.01			
Age	0.05	−0.03	−0.33**		
Attitudes towards vaccine efficacy	0.34**	0.16	0.13	0.28*	0.26**
Attitudes towards vaccine safety	0.12	0.13	0.01	0.24*	0.21**
Attitudes towards the danger of COVID-19 disease	0.06	0.07	−0.12	0.21*	0.17*
Attitudes towards COVID-19 susceptibility	0.13*	0.07	0.02	−0.05	0.06
Trust in societal factors	−0.02	0.09	0.20	−0.16	0.11
Social responsibility	0.24*	0.46**	0.25	0.35**	0.19**
*R*	0.73	0.85	0.63	076	0.74
*R* ^2^(adjusted)	0.52	0.70	0.35	0.55	0.54

* *P* < 0.05;

** *P* < 0.01.

## Discussion

This study is among the first research examining attitudes towards COVID-19 vaccination and the intention to get vaccinated against COVID-19 in Western Balkans countries, contributing to the large number of studies exploring COVID-19 vaccination intention worldwide.^6^^,^^18–20^ Almost half of the unvaccinated study participants did not intend to get vaccinated, while slightly more than one-third will probably or certainly get vaccinated in the future. Results of other studies showed that the potential acceptance rates of a COVID-19 vaccine ranged from over 90% in China, to <50% in Russia, Middle East, Africa and some European countries.^21^^,^^22^ In another study conducted in Bosnia and Herzegovina acceptance rate was 25.7%,^23^ while survey of essential workers in North Macedonia revealed acceptance rate of 44%.^24^ Survey conducted by UNICEF in Montenegro showed that COVID-19 vaccination acceptance rate was 53% in February 2021.^15^ Out of all mentioned countries, Serbia was the first to procure vaccines and consequently the intention to vaccinate was the least pronounced. At the time when study was conducted, unlike in other countries, in Serbia, the largest number of people who wanted to take vaccine had already been immunized. It should also be noted that possibly in some rural parts of surveyed countries people encountered difficulties regarding the access to vaccination points, resulting in their discouragement from vaccination.

Results of our study suggest that socio-demographic characteristics might not be particularly relevant factors of vaccination intention. Only younger age in Montenegrin sample was significantly associated with the stronger intention to get vaccinated against COVID-19. Similar to our findings, in a study conducted in Italy no significant association was found between socio-demographic variables (age, gender and employment status) and vaccination intention.^16^ In some other studies, willingness to get vaccinated against COVID-19 has been shown to be associated with factors, such as being male, an older adult, having a higher education. Overall, the association between socio-demographic factors and COVID-19 vaccination intention is highly heterogeneous between different countries, which can be attributed to cultural, socio-environmental and psychological factors.^17^

The results obtained in this study indicate that, in general, respondents in Serbia, Albania, Bosnia and Herzegovina, North Macedonia and Montenegro moderately believed that COVID-19 vaccine administration is the beneficial and safe intervention to successfully reduce the disease and that COVID-19 vaccine is harmless. Respondents in all five countries estimated that there is a moderately strong danger of COVID-19 in general, at the same time assessing that their personal risk to get sick was moderately high. It was observed that confidence in political and health authorities, science, and pharmaceutical companies was moderate, while personal sense of responsibility in achieving collective immunity and contagion prevention was moderately high (in Serbia, Albania, Bosnia and Herzegovina and Montenegro) and high (in North Macedonia).Our results obtained in Serbia, Bosnia and Herzegovina and Albania partly confirm previously established associations of vaccination intention with vaccination attitudes towards efficacy and safety, perceived risk of infection and disease severity.^21^^,^^22^^,^^25^ Similarly, main determinants of COVID-19 vaccine acceptance identified in Eastern European countries are public confidence in the vaccines’ safety and efficacy, besides institutional trust and health literacy.^14^ In general, the attitudes towards vaccine efficacy have been identified as one of the most important potential sources of vaccine hesitancy. Individuals who are uncertain that getting the vaccine will prevent the disease may understand vaccination as a risky behaviour since they cannot be confident that they will be effectively protected against the infection. People more inclined to believe in protective nature of vaccines are less likely to be vaccine hesitant.^26^^,^^27^ Vaccine safety has been identified as the most common concern regarding vaccination in other studies, too,^21^^,^^28^ and people more likely to believe in harmless nature of vaccines are less likely to be vaccine hesitant. Similar to our results, in previous studies respondents were more likely to be willing to get vaccinated against COVID-19 if they reported higher levels of perceived severity of COVID-19 disease.^29^^,^^30^ This finding implies that those who intend to get vaccinated view themselves as more likely to have significant consequences of the COVID-19 disease compared to those who do not intend to get vaccinated. On the contrary, in a study conducted in Saudi Arabia, 58% of respondents agreed that complications of COVID-19 can be very serious, but only 26.8% believed that they will be very sick if they get COVID-19.^31^ Having these various results in mind, it can be assumed that peoples’ perceptions of severity of COVID-19 are various and complex, and strongly associated with the vaccine behaviour.

Interestingly, in respondents from Serbia, Bosnia and Herzegovina, Macedonia and Albania social (collective) responsibility showed to be the strongest predictor of vaccination intention similarly to the studies from China and UK suggesting that collective responsibility (e.g. vaccinating oneself to help maintain herd immunity) may be facilitating vaccination uptake.^32^^,^^33^ Another study conducted in Bosnia and Herzegovina has similar results, with the intention to achieve collective immunity being the main reason for vaccination.^23^ Social responsibility refers to the awareness that people need to cooperate with each other for the benefit of a society at large,^34^ which is in this case collective immunity. Prosocial individuals generate adaptive and well-adjusted constructive responses to health and safety measures, and take care about the health and safety of others.^35^ This finding could be particularly valuable for public health campaigns. Vaccination attitudes are largely influenced by various socio-cultural, psychological and political factors.^5^ Therefore, there is a need to be sensitive on fears and hesitancy in the population, to recognize and understand them, and to address them in communication.

Although this study presents valuable evidence on COVID-19 vaccination intention in the population of Western Balkans, several limitations should be discussed. First, we sampled respondents through online channels, which excluded people who are not internet users. Online surveying was a method of choice since face-to face survey was not feasible due to the high rate of COVID-19 transmission during the study period. Second, convenience sampling does not provide sufficient level of representativeness. Third, since we employed cross-sectional design, causal relationships between variables cannot be established. Future studies with probability sampling and face-to-face distribution of questionnaires are needed to establish more general conclusions.

Results of this study suggest that vaccination interventions and campaigns aiming to improve the COVID-19 vaccine uptake should be focussed on specific set of factors in each country, promoting confidence in vaccine efficacy and safety and appealing to collective responsibility as most prevalent determinants of vaccination intention in Western Balkans. Interventions should be context-relevant, integrated, multi-component and based on risk communication, community engagement and social mobilization to increase trust and social cohesion resulting in increased COVID-19 vaccine acceptance among all stakeholders.

## Supplementary Material

ckad066_Supplementary_Data

## Data Availability

The data underlying this article will be shared on reasonable request to the corresponding author. The uptake of COVID-19 vaccines in Western Balkans countries is lagging behind the European Union average. Understanding factors that influence vaccine uptake in Western Balkans is critical for development of effective vaccination promotion strategies. Almost half of the respondents (46.8%) probably or certainly did not intend to get vaccinated against COVID-19. In most of the countries (Albania, Bosnia and Herzegovina, North Macedonia and Serbia), the strongest determinant of COVID-19 vaccination intention was the higher sense of social responsibility, while in Montenegro the age was the single predictor. Vaccination interventions should be focussed on specific set of factors in each country, appealing to social responsibility as most prevalent determinant of vaccination intention in Western Balkans.
