# Supplementary material for: Attitudes towards COVID-19 vaccination and intention to get vaccinated in Western Balkans: cross-sectional survey
Source: Eur J Public Health. 2023 Apr 29;33(3):496–501. doi: 10.1093/eurpub/ckad066 (PMC10234651; doi:10.1093/eurpub/ckad066)
Supplement: ckad066_Supplementary_Data [file ckad066_supplementary_data.docx]

**Table S1. Attitudes related to COVID-19 vaccination: scales and items**

| **Attitude scale** | **Items** |
| --- | --- |
| Attitudes toward COVID-19 vaccine efficacy | 1. In general, I think that vaccines against COVID 19 are effective.  2. Vaccination against COVID 19 will enable a return to normal life.  3. Vaccination is the only way to stop the COVID-19 pandemic.  4. This epidemic would last as long as it lasts, with or without vaccination.  5. There is not enough evidence that the COVID-19 vaccines really protect against the infection. |
| Attitudes towards COVID-19 vaccine safety | 1. Media allegations claiming the correlation between some of the vaccines against COVID 19 and the blood clots made me question the vaccine safety.  2. Claims of potential adverse effects of COVID-19 vaccines on fertility made me doubt the safety of this vaccine.  3.In general, I think that vaccines against COVID 19 are safe.  4. It is safer to get COVID-19 than to get vaccinated against it.  5. It has been proven that vaccines against COVID-19 contain substances that can harm human health.  6. The speed with which the vaccines were produced, made me question their safety. |
| Attitudes towards compulsory vaccination | 1. The state should not impose compulsory vaccination against COVID-19, but people should m.ake their own decisions on whether to be vaccinated.  2. Vaccination against COVID-19 should be mandatory for all citizens. |
| Attitudes towards danger of COVID-19 disease | 1. It is not necessary to get vaccinated against COVID-19, since it is an imaginary disease anyway.  2. The danger of COVID-19 is overstated.  3. COVID-19 does not give more severe clinical manifestations than seasonal flu.  4. I'm not the type of person who puts an effort into wearing a mask at all the places where it is recommended.  5. All the time during the epidemic, I try my best to maintain the recommended distance from others. |
| Attitudes towards COVID-19 susceptibility | 1. I believe I am immune to the corona virus (SARS-CoV-2).  2. I believe that the corona virus (SARS-CoV-2) is not very dangerous for me.  3. I'm scared that if I get sick with the COVID-19, I can have a very severe form of the disease. |
| Trust in societal factors | 1. Vaccination against COVID-19 is largely promoted by pharmaceutical companies in order to gain financial profits.  2. Pharmaceutical companies are reluctant to publish comprehensive and detailed research reports on the risks of adverse reactions to vaccines.  3. I believe that health authorities, when they encourage vaccination, do so with the best of intentions  4. I believe that political authorities, when they encourage vaccination, do so with the best of intentions.  5. Family physicians have an important role in educating people about the importance of vaccination against COVID-19.  6. I think that the principal motive for the scientists who participated in the creation of the vaccine against COVID-19 was profit. |
| Social responsibility | 1. I'm scared that if I get sick with the COVID-19 virus, I could transmit virus to others who could have a very severe form of the disease.  2. It is important that we all get vaccinated, in order to achieve collective immunity.  3. Since a sufficient number of people will be vaccinated, I don't think it is necessary for me to get the vaccine. |
